# Countering Antivax Misinformation via Social Media: Message-Testing Randomized Experiment for Human Papillomavirus Vaccination Uptake

**DOI:** 10.2196/37559

**Published:** 2022-11-24

**Authors:** Sunny Jung Kim, Jenna E Schiffelbein, Inger Imset, Ardis L Olson

**Affiliations:** 1 Health Behavior and Policy School of Medicine Virginia Commonwealth University Richmond, VA United States; 2 Massey Cancer Center Virginia Commonwealth University Richmond, VA United States; 3 Norris Cotton Cancer Center Geisel School of Medicine at Dartmouth Hanover, NH United States; 4 Department of Community and Family Medicine Geisel School of Medicine at Dartmouth Hanover, NH United States; 5 Department of Pediatrics Geisel School of Medicine at Dartmouth Hanover, NH United States

**Keywords:** misinformation, vaccine hesitancy, vaccine communication, social media, human papillomavirus, HPV, HPV vaccine

## Abstract

**Background:**

Suboptimal adolescent human papillomavirus (HPV) vaccination rates have been attributed to parental perceptions of the HPV vaccine. The internet has been cited as a setting where misinformation and controversy about HPV vaccination have been amplified.

**Objective:**

We aimed to test message effectiveness in changing parents’ attitudes and behavioral intentions toward HPV vaccination.

**Methods:**

We conducted a web-based message-testing experiment with 6 control messages and 25 experimental messages and 5 from each of the 5 salient themes about HPV vaccination (theme 1: safety, side effects, risk, and ingredient concerns and long-term or major adverse events; theme 2: distrust of the health care system; theme 3: HPV vaccine effectiveness concerns; theme 4: connection to sexual activity; and theme 5: misinformation about HPV or HPV vaccine). Themes were identified from previous web-based focus group research with parents, and specific messages were developed by the study team using content from credible scientific sources. Through an iterative process of message development, the messages were crafted to be appropriate for presentation on a social media platform. Among the 1713 participants recruited via social media and crowdsourcing sites, 1043 eligible parents completed a pretest survey questionnaire. Participants were then randomly assigned to 1 of the 31 messages and asked to complete a posttest survey questionnaire that assessed attitudes toward the vaccine and perceived effectiveness of the viewed message. A subgroup of participants (189/995, 19%) with unvaccinated children aged 9 to 14 years was also assessed for their behavioral intention to vaccinate their children against HPV.

**Results:**

Parents in the experimental group had increased positive attitudes toward HPV vaccination compared with those in the control group (t_969_=3.03, *P*=.003), which was associated with increased intention to vaccinate among parents of unvaccinated children aged 9 to 14 years (*r*=1.14, *P*=.05). At the thematic level, we identified 4 themes (themes 2-5) that were relatively effective in increasing behavioral intentions by positively influencing attitudes toward the HPV vaccine (*χ*^2^_5_=5.97, *P*=.31, root mean square error of approximation [RMSEA]=0.014, comparative fit index [CFI]=0.91, standardized root mean square residual [SRMR]=0.031). On the message level, messages that provided scientific evidence from government-related sources (eg, the Centers for Disease Control and Prevention) and corrected misinformation (eg, “vaccines like the HPV vaccine are simply a way for pharmaceutical companies to make money. That isn’t true”) were effective in forming positive perceptions toward the HPV vaccination messages.

**Conclusions:**

Evidence-based messages directly countering misinformation and promoting HPV vaccination in social media environments can positively influence parents’ attitudes and behavioral intentions to vaccinate their children against HPV.

## Introduction

### Human Papillomavirus Vaccination in the United States

Human papillomavirus (HPV) is a group of >200 viruses that can infect all genders [1]. HPV infection can cause 6 types of cancer, including cancers of the penis, vulva, cervix, vagina, anus, and oropharynx [1]. The HPV vaccine Gardasil 9 protects against infection with 7 high-risk types of HPV that cause cancer and 2 types that cause genital warts [2]. Within 6 years after the HPV vaccination became available, studies found that the HPV vaccine had lowered the rate of cancer-causing HPV infections by 64% and the risk of cervical precancer by 47% [3]. According to a report by the Centers for Disease Control and Prevention (CDC) published in 2018, a total of 92% of HPV-associated cancers can be prevented by the HPV vaccine [4]. Despite evidence that the HPV vaccine is highly effective in preventing cancers caused by HPV infections, the percentage of adolescents starting the vaccine series in the United States is lower compared with the percentage receiving other routinely recommended vaccines [5]. Only 51% of teens in the United States received the full HPV vaccine series in 2018, which is far lower than that in other industrialized countries, such as the United Kingdom and the Healthy People 2020 goal of 80% [6-8].

### Vaccine Hesitancy and Misinformation on Social Media

Since the HPV vaccine was approved in 2006, the prevalence of misinformation on social media as well as the lack of educational campaigns regarding HPV vaccines have contributed to HPV vaccine hesitancy and refusal [9-11]. Within the first 10 years since the approval of the vaccine, nearly 40% of Facebook posts about the HPV vaccine contained texts, links, and images that amplified the perceived risks of the HPV vaccine [12]. Studies have reported that parents’ knowledge and HPV vaccine hesitancy are influenced by various factors and media sources, including repeated exposure to misinformation on social media [2,13,14]. One study [15] found that myths and safety concerns about the HPV vaccine were widespread on Twitter, and Twitter users were more likely to be exposed to tweets reporting safety concerns than tweets supporting the evidence of HPV vaccine effectiveness and safety. When looking at aggregated Twitter data, researchers found that vaccination rates were lower in the United States, where tweets on negative representations of vaccines, misinformation, and conspiracies were most prevalent [16]. Parents who declined to vaccinate their children against HPV were shown to mistrust health care systems and have negative attitudes toward HPV vaccination [11]. These psychological states, resulting from repeated exposure to misinformation about HPV vaccination, contribute to parents’ resistance and hesitancy to follow the HPV vaccine recommendation [17]. Effectively addressing misinformation is critical for resolving vaccine hesitancy and refusal [16,18].

### Health Communication on Social Media for HPV Vaccine Uptake

Health communication and education programs play a vital role in countering misinformation regarding HPV vaccines. According to the CDC, strategic mass media communication is one of the best approaches to addressing a specific public health issue [19,20]. Web-based platforms offer a cost-effective outlet to share, convey, and disseminate health information without geographic constraints and can be leveraged to engage hard-to-reach populations and monitor health behaviors in real time [2]. Despite the potential public health impact and benefits of public health messages that can be promoted on social media, a substantial knowledge gap exists in how to design, examine, and identify effective messages for social media campaigns that are persuasive in promoting HPV vaccination uptake. Given the effect of misinformation on eliciting vaccine hesitancy, it is critical to develop and evaluate accurate and persuasive social media campaign messages.

In this study, we compared different message themes related to HPV vaccine hesitancy and refusal and examined the themes and messages that were more effective in promoting HPV vaccine uptake. We hypothesized that exposure to HPV vaccine uptake messages will influence parents’ behavioral intention to vaccinate their children against HPV by improving their attitudes toward the vaccine.

## Methods

### Participant Recruitment and Eligibility

We recruited participants through paid advertisements on Facebook and Amazon’s Mechanical Turk (MTurk). MTurk is a web-based labor market with more than 500,000 anonymous workers worldwide, where requesters distribute tasks and registered workers complete them. MTurk has been widely used for various research purposes such as surveys, cognitive tasks, and web-based experiments [21,22]. Prospective participants who viewed our recruitment messages on MTurk or Facebook were redirected to a secure survey platform for consent and eligibility screening. In our screening survey, we assessed participant characteristics such as ZIP code, sex, age, and gender of children. To be eligible, prospective participants needed to be residents of the United States, have at least 1 child, and provide their ZIP code and state of residence. Eligible participants who provided consent were then directed to the message-testing experiment.

### Message-Testing Experiment

Once directed to the message-testing experiment, participants completed a pretest survey (see Multimedia Appendix 1 for survey instruments) that assessed baseline attitudes and knowledge about HPV and HPV vaccination as well as their behavioral intention to vaccinate their children against HPV. After completing the pretest survey questions, participants were randomly assigned to 1 of 31 messages (25 experimental messages and 6 control messages), viewing 1 message per participant. All the stimulus messages were presented in a simulated social media environment (see Multimedia Appendix 2 for messages). To experimentally control for high elaboration likelihood (eg, processing information carefully rather than heuristically), participants were asked to view the assigned message carefully and report what they read and viewed [23]. After exposure to the assigned message, participants were asked to complete the posttest surveys. When the participants completed the posttest survey, they received educational resources on HPV vaccination.

### Message Development

We conducted a series of virtual focus groups with parents to identify the key barriers and reasons for HPV vaccine hesitancy and refusal (results reported elsewhere) before this study. Through focus group discussions, we identified 5 salient themes to address in our messages on HPV vaccination.

1. Safety, side effects, risk, and ingredient concerns and long-term or major adverse events
2. Distrust of the health care system
3. HPV vaccine effectiveness concerns
4. Connection to sexual activity
5. Misinformation about HPV or HPV vaccine

We developed 5 experimental messages for each theme and 6 control messages on e-cigarettes. The experimental messages were grounded in scientific information from published articles and reputable health organizations (eg, CDC, National Cancer Institute, and American Cancer Society). The experimental messages were designed to help the audience understand HPV and HPV vaccination and included a call to action to vaccinate their children against HPV.

### Message Framing

Message-framing tactics were applied to change parents’ attitudes and behavioral intentions toward HPV vaccination. To avoid confounding effects, we controlled for elements that were not part of the experimental manipulation designs. First, we controlled the length of the messages to no more than 130 words and the message source across all 31 messages to be identical and neutral (ie, the same Facebook account appeared as the poster of the simulated Facebook messages). Second, we controlled the message structure: each message started with introductory statements, included tailored statements specific to the message theme, provided a photo or link to a video that was congruent with the message, and ended with the same call to action. We countered common myths and misinformation and added the correct information. This message structure is consistent across all stimulus messages.

The research team conducted multiple iterative review processes with content experts in the fields of adolescent health, cancer prevention, and health communication before finalizing the messages (Multimedia Appendix 2). Through a web-based pre-post randomized message-testing experiment, we examined the persuasion effects of these messages on changing parents’ attitudes toward the vaccine and their behavioral intention to vaccinate their children against HPV.

### Measures

#### Pretest Survey

Eligible participants were asked to answer pretest questions assessing their baseline knowledge of and attitudes toward HPV and HPV vaccination, response efficacy beliefs, and behavioral intention to vaccinate their children against HPV. For response efficacy, we developed items that measured the degree to which participants thought the HPV vaccine was effective in preventing cervical cancer and mouth or throat cancer on a 5-point Likert scale (1=not at all effective; 5=extremely effective) with an additional option indicating “I don’t know.” To measure attitudes toward HPV vaccination, we used 9 items from a study by Kim and Niederdeppe [24] and modified them to assess parents’ attitudes toward the HPV vaccine on the 7-point semantic differential scale, including measures of *bad-good*, *harmful-beneficial*, *useless-useful*, and *unsafe-safe* (see Multimedia Appendix 1 for the full list of survey items).

To assess the vaccination status of their children against HPV, we first asked, “Do you have any sons (or any daughters)?” Among parents who indicated having sons or daughters, we asked “How old are your sons (or daughters)? Please select all that apply.” Multiple choice options were available to indicate the ages of sons or daughters (“less than 5 years old,” “5 to 8 years old,” “9 to 14 years old,” “15 to 18 years old,” and “older than 18 years old).” For those who reported having sons or daughters aged between 9 and 14 years, we asked, “Has your 9-14 year-old son (or daughter) been vaccinated for HPV? If you have more than one son (or daughter) 9-14 years old, please answer about the son (or daughter) who had the most recent birthday.” Four response options were available, including “Yes, s/he has received two or more HPV vaccine shots,” “Yes, s/he has received one HPV vaccine shot,” “No, s/he has not received any HPV vaccine shots,” and “I don’t know.” For parents who indicated not receiving any HPV vaccine shots or “I don’t know,” we displayed the following behavioral intention item on a 5-point Likert scale, “Thinking about the same 9-14 years old son (or daughter), how likely is it that s/he will receive the HPV vaccine in the next 12 months?” The response options were 1=very likely and 5=very unlikely. Greater values indicate a lower intention to not vaccinate their unvaccinated children against HPV.

#### Posttest Survey

During the posttest survey, in addition to remeasuring the items in the pretest survey, such as attitudes toward HPV vaccination, participants were guided to answer a series of additional posttest items, including manipulation check questions to ensure participants had viewed the messages, as well as message perceptions. To understand message perceptions, we adapted 5 items from the message sensation value scale [25] and 4 items from the perceived message effectiveness scale [26] and modified their wording to fit into the study context. Message sensation value, the degree to which message features elicit affective and arousal responses, was assessed on a 7-point Likert scale, and perceived message effectiveness was measured on a 5-point Likert scale. These 2 questionnaires were reported separately.

An identical behavioral intention question was displayed for a subgroup of parents who indicated at pretest having unvaccinated children aged 9 to 14 years (see Multimedia Appendix 1 for survey measures).

### Ethical Considerations

All study procedures were reviewed and approved by the Committee for the Protection of Human Subjects (the institutional review board) at Dartmouth College, and the institutional review board reliance has been approved by Virginia Commonwealth University and Dartmouth College (HM20014090 and MOD00009013, respectively). The study team obtained a waiver for documenting participant signatures during the consenting process; an information sheet about the study was provided to prospective participants, and they were asked to click *next* to provide their consent and continue to participate in the study. After a trained researcher verified worker IDs entered into the survey, participants recruited from MTurk who completed the experiment were compensated at US $1.80. Given the length of our experiment, this compensation rate on MTurk was acceptable, as the average compensation rate was 10 cents per minute. Worker IDs were deleted and not linked to any of the study data before analysis. Participants recruited from paid advertisements on Facebook had a chance to enter a drawing to receive one of 50 e-gift cards each worth US $10. To protect the confidentiality of participants who were recruited via Facebook paid advertisements, the study team did not collect participant names and only collected email addresses for the purposes of compensation administration; those email addresses were collected, stored, and managed securely separately from the study data.

### Data Analysis

We first conducted descriptive statistics on demographic variables with the sample and by comparing experimental and control groups. These subgroup comparisons were conducted for pretest variables, including prior knowledge and response efficacy beliefs measures. Mean-based composite scores were generated to assess attitudes toward HPV vaccination on the pretest (Cronbach α=.96; mean 4.98, SD 1.50) and posttest survey items (Cronbach α=.96; mean 5.11, SD 1.47). The attitude change scores were calculated by subtracting the pretest composite values from the posttest composite values for each participant (mean difference 0.12, SD 0.92). Greater values indicate more positive changes in attitudes toward HPV vaccination. Five posttest items assessing the sensation value of the viewed messages on a 7-point Likert scale were averaged to form a composite score (Cronbach α=.92; mean 4.57, SD 1.50). Greater values indicate a more positive sensation toward the messages. A total of 4 posttest items measuring perceived message effectiveness on a 5-point Likert scale were averaged to form a composite score (Cronbach α=.80; mean 3.29, SD 0.99). Greater values indicate a greater perceived effectiveness of the message.

For thematic-level analyses, a path-modeling approach was used to model persuasion pathways from message exposure to attitudinal changes toward HPV vaccination, which indirectly influenced behavioral intentions to vaccinate. For the eligibility of thematic-level analyses, which included behavioral intention measures for HPV vaccination in the next 12 months measured at the posttest survey, we only included a subset of data from 189 parents with children aged between 9 and 14 years who had not received any HPV vaccine shot. We used 5 dummy variables to generate exogenous constructs in the path model. The control condition was used as the reference group. Path coefficients from dummy-coded variables to the attitudinal change construct were the magnitude of the associations compared with those from the reference group (control group).

For individual message-level analyses, we used the ANOVA with a Bonferroni correction within each theme. Composite scores of measures assessing attitudes toward HPV vaccination, sensation values of the viewed message, and perceived message effectiveness were evaluated in the ANOVA framework to statistically identify relatively more effective messages within each of the 5 thematic blocks.

## Results

### Participants

**Figure 1:**
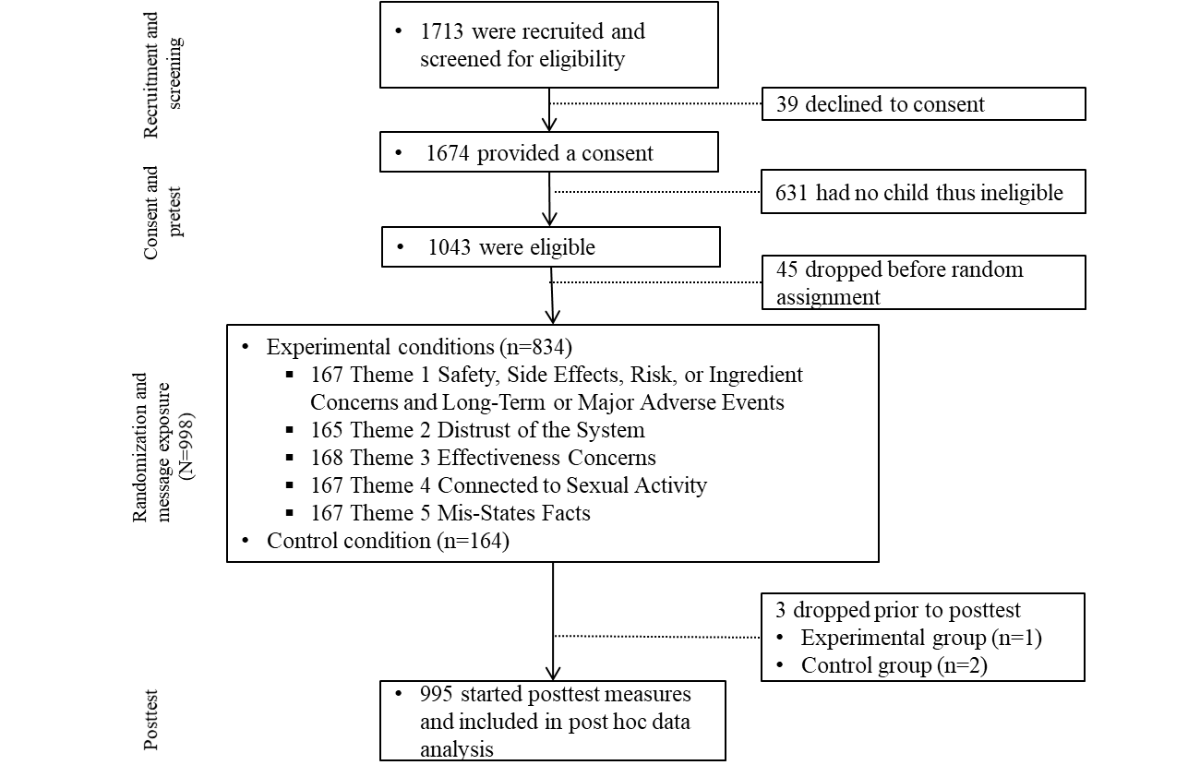
CONSORT (Consolidated Standards of Reporting Trials) diagram showing the flow of participants through pre-post message-testing experiment.

A total of 1674 respondents provided consent and participated in the eligibility screening survey, and 1043 (62.31%) were eligible to participate in the study. Among the 1043 participants, 45 (4.31%) left the study before being randomized to a message, and an additional 3 participants left during the posttest questionnaire, leading to a final sample size of 998 for pretest data analysis and 995 for the posttest data analysis (Figure 1). The average age was 35.39 (SD 10.58) years; 616 (61.7%) were female; 202 (37.5%) had an annual income of US ≤$50,000 (see Table 1 for demographic characteristics of our participants).

**Table 1:** Baseline characteristics by the assigned group (N=998).

Characteristics		Experimental group (n=834)	Control group (n=164)	Total (N=998)
Age (years), mean (SD)		37.24 (10.5)	34.77 (10.5)	35.39 (10.6)
Female, n (%)		520 (62.4)	96 (58.5)	616 (61.7)
**Income**				
	<US $50,000, n (%)^a^	172 (38.6)	30 (32.3)	202 (37.5)
**Education**				
	No college, n (%)	58 (13.1)	16 (17.2)	74 (13.8)
Daughter (yes), n (%)		653 (78.3)	126 (76.8)	779 (78.1)
Son (yes), n (%)		585 (70.1)	125 (76.2)	710 (71.1)
Rural, n (%)		180 (22.4)	36 (23.2)	216 (22.6)
**Previous knowledge at pretest**				
	Heard of HPV^b^? (Yes), n (%)^c^	730 (88.1)	142 (87.1)	872 (87.9)
	Heard of the HPV vaccine? (Yes), n (%)^d^	691 (83.3)	136 (83.4)	827 (83.3)
Perceived importance^e^ of HPV vaccines, mean (SD)		2.44 (1.3)	2.34 (1.4)	2.42 (1.3)
**Response efficacy^f^ at pretest of HPV vaccine**				
	In preventing cervical cancer? (Do not know), n (%)	118 (14.1)	22 (13.4)	140 (14)
	In preventing mouth or throat cancer? (Do not know), n (%)	234 (28.1)	53 (32.3)	287 (28.8)
	In preventing cervical cancer? mean (SD)	2.53 (1.6)	2.48 (1.5)	2.52 (1.6)
	In preventing mouth or throat cancer? mean (SD)	2.26 (1.8)	2.08 (1.8)	2.23 (1.8)

^a^Education and income were assessed in the later phase of the experiment after posttest measures; thus, the sample size for these socioeconomic measures was smaller (n=539 and n=536 for reporting income and education, respectively).

^b^HPV: human papillomavirus.

^c^N=992 for this row because 6 people did not answer this question.

^d^N=993 for this row because 5 people did not answer this question.

^e^The perceived importance of vaccinating their children against HPV was measured on a 5-point Likert scale, where 1=extremely important and 5=not important.

^f^Response efficacy belief at baseline=greater values indicate stronger belief in response efficacy.

### Outcomes on Pretest Measures

Nearly 88% (872/992, 87.3%) of the participants reported having heard of HPV before the study, and 83.3% (827/993) reported that they had heard of the HPV vaccine. At the pretest survey, 14% (140/998) of participants indicated that they did not know about the efficacy of the HPV vaccine in the prevention of cervical cancer, and 28.8% (287/998) of participants did not know of the efficacy of the HPV vaccine in preventing mouth or throat cancer (Table 1).

### From Message Exposure to Behavioral Intention to Vaccinate

At a group level (experimental group vs control group), experimental messages about the HPV vaccine significantly increased positive attitudes toward HPV vaccination compared with the control messages about e-cigarettes (t_969_=3.03, *P*=.003). Parents’ behavioral intention to vaccinate their children against HPV was significantly associated with the increase in positive attitudes toward HPV vaccination (B=1.14, *P*=.05).

#### Thematic-Level Outcomes

**Figure 2:**
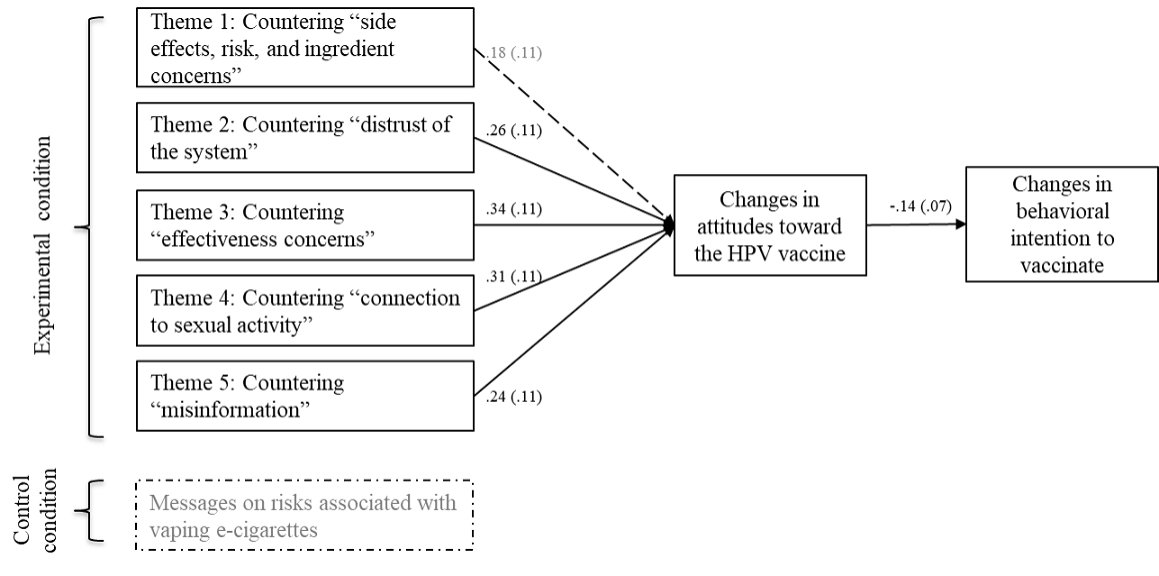
Thematic-level path analysis. HPV: human papillomavirus.

At the thematic level, according to the results from path modeling, messages countering 4 themes—theme 2: distrust of the health care system, theme 3: HPV vaccine effectiveness concerns, theme 4: connection to sexual activity, and theme 5: misinformation about HPV or HPV vaccine—were more likely to increase behavioral intention to vaccinate, in part because of the increased positive attitudes toward the vaccine (*χ*^2^_5_=5.97, *P*=.31*,* root mean square error of approximation [RMSEA]=0.014, comparative fit index [CFI]=0.91, standardized root mean square residual [SRMR]=0.031). Figure 2 denotes thematic-level path analysis results from message exposure to changes in behavioral intention among parents with unvaccinated children aged 9 to 14 years (n=189). Greater values of the changes in behavioral intention in Figure 2 indicate lower behavioral intention to vaccinate. A dotted line in Figure 2 indicates a nonsignificant path. Straight lines with standardized path coefficients are at the 0.05 level of significance. Greater values to the attitudes construct indicate more positive attitudinal changes toward the HPV vaccine after viewing the message.

#### Message-Level Outcomes

Within each theme, the influence of each message on changing vaccine attitudes, sensation values of the message viewed, and perceived message effectiveness were examined. For example, at the individual message level, compared with message IDs b, d, and e in theme 1, the message ID c in theme 1, which directly countered safety concerns on the HPV vaccine with an educational video that addressed HPV vaccine effectiveness, significantly increased the sensation value (eg, positive attitudes) toward the message effectiveness (mean 5.38, SD 1.07, *F*_4,160_=6.59, *P*<.01) and perceived message effectiveness (mean 3.79, SD 0.83, *F*_4,160_=4.06, *P*=.004; Table 2). Within theme 2, the message ID b (mean 5.07, SD 1.31) countering a myth about a pharmaceutical money-making scheme (eg, “vaccines like the HPV vaccine are simply a way for pharmaceutical companies to make money. That isn’t true”) was significantly more effective in increasing positive attitudes toward the message than the message ID c (mean 3.90, SD 1.76) that countered a myth about a doctor money-making scheme (eg, “We know that some people worry that vaccines like the HPV vaccine are just helping doctors make money”; *F*_4,158_=3.01, *P*=.02). Within theme 3 countering HPV vaccine effectiveness concerns, the message ID c (mean 3.65, SD 0.92) reporting statistical evidence of the HPV vaccine’s effectiveness in preventing cancer (eg, “...among women who were vaccinated in Finland 15 years ago, none of them got HPV cancers” and “...we have seen a 71% decrease in HPV infections that cause most HPV cancers and genital warts among teen girls vaccinated in the U.S.”) was perceived more effective than the message ID a (mean 2.91, SD 1.08) that stated the importance of HPV vaccination to effectively treat HPV infections (eg, “The HPV vaccine does not treat HPV infections that already exist, though, and there is no treatment for HPV infections. That’s why it’s so important for boys and girls to get vaccinated before they can ever get exposed to HPV infections”), *F*_4,161_=3.32, *P*=.01. Within theme 4, although the ANOVA test reported that the mean values of the 5 messages differed (*F*_4,160_=3.01, *P*=.02), the significance did not emerge when a Bonferroni correction was applied. For theme 5, which focused on countering misinformation about HPV or HPV vaccination, the message ID e (mean 3.79, SD 0.72) correcting misinformation about HPV vaccine recommendations (eg, “Why both boys and girls? Because everyone can get HPV cancers caused by HPV” and “Why two doses? For full protection against approximately 93% of HPV cancers, more than one vaccine dose is needed”) was more persuasive than the message ID d (mean 3.06, SD 1.01) addressing the misinformation that the HPV vaccine can cause ovarian failure and fertility issues (*F*_4,159_=3.07, *P*=.02).

**Table 2:** Results from ANOVA tests at the message level with a Bonferroni correction. Italicized values indicate a thematic block with a significant difference less than 0.05 before a post hoc test as shown in *F* values in ANOVA. The significance of italicization level is noted next to the *F* value within the block.

Themes and message IDs			Changes in attitudes toward HPV vaccination, mean (SD)		Perceived sensation value toward messages, mean (SD)		Perceived message effectiveness, mean (SD)
**Theme 1 (n=162-165)**					*F*_4,160_=6.59, *P*<.001		*F*_4,160_=4.06, *P*=.004
	a	−0.10 (0.84)		*4.69* ^e^ *(1.42)*		*3.41 (0.98)*	
	b	−0.09 (0.61)		*4.29* ^c^ *(1.44)*		*3.19 (1.02)*	
	c	0.03 (0.89)		*5.38* ^b,e^ *(1.07)*		*3.79* ^e^ *(0.83)*	
	d	0.19 (0.62)		*4.18* ^c^ *(1.68)*		*3.35 (1.01)*	
	e	0.40 (0.92)		*3.66* ^a,c^ *(1.50)*		*2.85* ^c^ *(1.05)*	
**Theme 2 (n=163)**					*F*_4,158_=3.01, *P*=.02		Not significant
	a	0.27 (0.73)		*4.64 (1.52)*		3.38 (1.07)	
	b	0.31 (1.17)		*5.07* ^c^ *(1.31)*		3.45 (0.88)	
	c	−0.003 (0.71)		*3.90* ^b^ *(1.76)*		3.12 (1.12)	
	d	0.16 (0.63)		*4.28 (1.15)*		2.99 (0.96)	
	e	0.06 (0.83)		*4.37 (1.38)*		3.19 (0.99)	
**Theme 3 (n=165-166)**					Not significant		*F*_4,161_=3.32, *P*=.01
	a	0.12 (0.81)		4.27 (1.48)		*2.91* ^c,d^ *(1.08)*	
	b	0.24 (0.87)		4.93 (1.34)		*3.44 (0.83)*	
	c	0.34 (1.06)		4.92 (1.46)		*3.65* ^a^ *(0.92)*	
	d	0.33 (1.14)		4.91 (1.39)		*3.55* ^a^ *(0.70)*	
	e	0.10 (0.82)		4.78 (1.44)		*3.30 (0.99)*	
**Theme 4 (n=165)**					*F*_4,160_=3.01, *P*=.02		Not significant
	a	0.24 (1.23)		*4.33 (1.35)*		3.48 (0.95)	
	b	0.17 (0.68)		*5.09 (1.67)*		3.54 (1.12)	
	c	0.44 (0.95)		*5.09 (1.44)*		3.79 (0.95)	
	d	0.03 (0.71)		*4.09 (1.65)*		3.16 (1.1)	
	e	0.14 (0.75)		*4.61 (1.24)*		3.24 (0.7)	
**Theme 5 (n=163-164)**					Not significant		*F*_4,159_=3.07, *P*=.02
	a	0.004 (1.06)		4.14 (1.54)		*3.29 (0.90)*	
	b	0.37 (1.67)		4.59 (1.62)		*3.36 (1.10)*	
	c	−0.03 (1.02)		4.48 (1.57)		*3.15 (0.93)*	
	d	0.04 (0.85)		4.12 (1.36)		*3.06* ^e^ *(1.01)*	
	e	0.29 (0.68)		4.98 (1.47)		*3.79* ^d^ *(0.72)*	

^a,b,c,d,e^Message IDs with a significant difference after Bonferroni correction were reported. Superscript letters indicate a significant difference (*P*<.05) against the message ID in the row within each theme. Theme 1: safety, side effects, risk, and ingredient concerns and long-term or major adverse events. Theme 2: distrust of the health care system. Theme 3: HPV vaccine effectiveness concerns. Theme 4: connection to sexual activity. Theme 5: misinformation about HPV or HPV vaccine. Greater values indicate more positive changes in attitudes toward HPV vaccination, greater sensation and positive attitude toward the message viewed, and greater perceived effectiveness of the message viewed.

## Discussion

### Principal Findings

Findings from path modeling confirmed that HPV messages can yield positive directions in changing attitudes and behavioral intentions toward the HPV vaccine. Our path model indicated that most of the themes were effective in significantly changing parents’ attitudes toward HPV vaccination, which, in turn, was strongly associated with behavioral intention to vaccinate their children against HPV. Those effective communication themes were targeting the distrust of the health care system by correcting misinformation (eg, “doctors/pharmaceutical companies make money-scheme”), addressing HPV vaccine effectiveness concerns, educating on perceived connection to sexual activity, and correcting common myths about HPV vaccination. Given that parental views are often altered because of misinformation that is easily accessible on social media or web-based communities, our study demonstrates that evidence-based messages that directly correct misinformation may be effective in enhancing public knowledge and attitudes about HPV vaccination.

As shown in the ANOVA results, certain messages within each thematic block were more effective than other messages in generating positive perceptions of the message viewed. For example, messages that provided numeric evidence from credible sources (eg, “As you will hear in this video from the Minnesota Department of Health...”) were perceived more persuasive than messages that did not refer to a credible source (eg, “The vaccine’s safety has continued to be studied in the 12 years...”). It should also be noted that there was no message that generated adverse effects or boomerang effects of persuasion, such as stimulating the opposite stance [27,28].

We acknowledge that attitudes and behavioral intentions toward HPV vaccination are likely to differ according to the level of issue involvement [29], the HPV vaccination status in this case. Thus, in our path model, we included only a subgroup of parents with unvaccinated children aged 9 to 14 years (n=189). Thus, findings based on the path model should be specific to the parents of unvaccinated children in this age group. However, it should be noted that the analyses of constructs related to message evaluations (ie, message sensation and perceived message effectiveness) were based on the full sample of parents. Exposure to media and campaign messages is conceptualized as distal predictors of behavioral changes according to the Integrative Model of Behavioral Prediction [30]. Evaluations of messages from parents who had already vaccinated their children could be different from those of parents who have not yet vaccinated their children. Although one’s issue involvement status (ie, whether vaccinated) could influence a parent’s attitudes and behavior intentions toward HPV vaccination, in this work regarding perceptions about the messages, we do not have either predefined theoretical justification or statistical power to gauge whether message sensation and perceived message effectiveness will differ by children’s vaccination status. Future studies should examine whether children’s HPV vaccination status will influence parents’ perceived message sensation and perceived message effectiveness.

The prevalence of myths and misinformation about HPV and HPV vaccination on social media influences parents’ decisions about HPV vaccination [31,32]. Our study empirically examined the persuasion effects of messages that were strategically designed to counter misinformation about HPV vaccines while promoting HPV vaccination in social media environments. As evidenced in the path model, we demonstrated that systematic designs of communication themes and message components not only influence overall parental attitudes and evaluations of the messages but also can positively change behavioral intention toward HPV vaccination for unvaccinated children. Accumulating evidence suggests that digital technologies, including social media, are ubiquitous in promoting public health, even among often hard-to-reach populations such as rural residents [33]. Our study indicates that social technologies can be leveraged to deliver strategic public health communications to people who may have limited health care resources to intervene in their behaviors and improve their attitudes toward the HPV vaccine [9,22].

Parents’ hesitancy toward vaccinating their children against HPV was, in part, because of psychological barriers, such as skepticism about HPV vaccine effectiveness and distrust toward the health care system, which was influenced by misinformation about HPV vaccination. On the basis of the salient themes that emerged as parents’ reasons not to follow HPV vaccine recommendations, most of the themes we used in our message designs focused on addressing the lack of knowledge and misunderstandings about HPV vaccine recommendations. For example, we developed messages that directly countered the misinformation that the HPV vaccine is lucrative for health care systems and pharmaceutical industries, which had led some parents not to follow the HPV vaccine recommendations. We also developed multiple messages that directly countered misinformation regarding the side effects and safety issues associated with the HPV vaccine. This type of misinformation has been prevalent on social media, which has been a contributing factor in vaccine refusal and hesitancy among parents [34,35].

It should be noted that our parent sample was slightly younger, more educated, and poorer than the nationwide samples of parents reported by the Pew Research Center’s Internet and American Life Project and the US Census Bureau Social and Economic Supplement Surveys [36,37]. For example, among those who reported their income, 37.5% (202/539) of our participants had annual incomes lower than US $50,000, whereas in the 2018 Annual Social and Economic Supplement survey, 32.1% of households with children ≤18 years had an annual income lower than US $50,000 in 2017 [37]. However, given that our study was an experiment examining internal validity, random sampling was not the primary concern in designing the study. Rather, our primary goals centered on ensuring random assignment, assessing the status of the theoretical explanations, and testing whether the findings will replicate in other settings. Our primary goals of the experimental study were to “apply the theory beyond the research setting” and “the degree to which the specific sample represents the population of interest was of less importance” as emphasized in Highhouse [38].

In fact, multiple replication studies support the utility of web-based samples such as MTurk for experimental studies. Through a series of 15 replication experiments comparing results from MTurk convenience samples to probability samples, Coppock [39] confirmed that estimates of causal effects obtained on MTurk samples were similar to those obtained on probability-based national samples. Mullinix et al [40] replicated 20 experiments and found a high level of concordance between estimates obtained from MTurk-based samples and national probability samples. Along with the supporting evidence confirming the utility of web-based convenience samples in experimental studies, we carefully implemented several methods to improve our ability to generalize causal inferences across different settings and contexts. First, to make our findings relevant and generalizable, we generated stimulus materials that reflect social media posts that people would encounter in their real life and conducted the experiment in a real-world setting. These stimuli were designed to simulate the way people view, read, and process social media posts. Second, we conducted formative research to generate the most salient campaign themes and used multiple instances of a stimulus category (HPV vaccine-related posts on social media) to ensure that the experimental stimuli were representative of a predefined population of stimuli (ie, social media posts related to HPV vaccine and misinformation) while controlling for possible third variables (eg, by having the length of the messages consistent across stimuli and using the same source profile across stimulus materials).

Our study methods were designed to enhance our understanding of the theory-based causal process (eg, how parents process social media messages countering misinformation and promoting HPV uptake) and ecological validity (ie, “the realism of the experimental methods, materials, and settings” [38]. Given the relatively small size of the subgroup sample in the path model, we have implications of the path model findings only for parents with unvaccinated children aged 9 to 14 years who use the web, not generalizing across subpopulations of parents with specific background factors.

It should be noted that neither external nor internal validity can be captured through a single experiment. In addition, as Lin et al [41] argue, there may be a trade-off between internal and external validity. We posit that the external validity of our findings should be examined via replication. Moreover, when a number of studies on this topic become available, conducting a meta-analysis of the causal process will help examine whether the message effects found in our study remain reliable across the results of many studies. As per concerns related to generalizability, future research should further examine whether the message effect is moderated by interactions with one or more demographic factors (parents of children in different age groups). A strong replication pattern across samples will improve the credence of the findings.

### Limitations

Our findings should be interpreted in light of the limitations imposed by the study designs and settings. First, the experiment was based on a pre-post design that measured message effects without a time lag or long-term follow-up; that is, although our message-testing experiment used random assignment to a stimulus message to build internal validity, perceived message effectiveness and sensation values (eg, attitude toward the messages) measured after message exposure could be driven by immediate recency effect and priming effect that can diminish over time [42]. To measure the long-term effects of message exposure, a separate condition that entails a pre-post design with a long-term follow-up should be implemented to rule out the possible immediate recency and priming effects of message exposure. Future research should also examine the impact of social media campaign messages on vaccination behavior during follow-up assessments.

Second, for our path analyses, we generated dummy-coded exogenous variables to examine persuasion pathways from the conditions (eg, experiment condition=1 and control condition=0) and also from the 5 thematic-level variables, as shown in Figure 2. However, we did not compute dummy-coded variables for the message-level constructs in the path model. This finding was partially due to the sample size. If dummy coding were generated at the message level, the model would entail 25 exogenous variables, leading to overfitting problems and unspecified model fits. To resolve the issue, we instead conducted composite score–based tests using ANOVA with a Bonferroni correction. This approach helped specify the exact message units that have shown effects on persuasion outcomes. In this study, although we used a pre-post randomization experiment as the most suitable design for between-subject tests across conditions and thematic-level analyses, we suggest that future research should consider implementing a within-subject discrete choice experiment [9] to test prespecified message attributes, such as types of message sources and pictorial vs moving images.

Finally, although most of our analyses were based on a sample of over 900 participants, the final path model was based on a subset of the sample (n=189) with children aged between 9 and 14 years, who did not receive any HPV vaccines at the moment, to reflect the age range for the HPV vaccination most recommended by the CDC; that is, we did not ask a hypothetical question to all participants (eg, “*If* you had a child aged between 9 and 14, would you consider HPV vaccination in the next 30 days?”). Instead, we asked about the true behavioral intention for HPV vaccination only among parents with children aged between 9 and 14 years who had not yet vaccinated their children against HPV (189/995, 19%). Future experimental research may replicate the path analysis and further test potential moderators or mediators discussed in the literature. Identifying moderators and mediators involved in the persuasion pathway for HPV vaccination uptake will help to identify effective communication tailoring strategies.

### Conclusions and Public Health Implications

Our study highlights that strategic communication efforts in social media environments can offer the opportunity to change parental attitudes and behavioral intentions toward HPV vaccination. Given how ubiquitous social media platforms are today, promoting evidence-based messages—such as ours—on social media may play an important role in promoting accurate health information and enhancing knowledge and attitudes about HPV and HPV vaccination for cancer prevention. We found that when harnessing social media platforms for public health communications, directly countering dominant misinformation themes and providing accurate science-based information can be particularly effective in promoting HPV vaccination uptake.

## Appendix

Multimedia Appendix 1 Pretest and posttest survey items.

Multimedia Appendix 2 Stimulus messages for the experimental group.

## Abbreviations

CDC: Centers for Disease Control and Prevention
CFI: comparative fit index
HPV: human papillomavirus
MTurk: Mechanical Turk
RMSEA: root mean square error of approximation
SRMR: standardized root mean square residual
