# Supplementary material for: Countering Antivax Misinformation via Social Media: Message-Testing Randomized Experiment for Human Papillomavirus Vaccination Uptake
Source: J Med Internet Res. 2022 Nov 24;24(11):e37559. doi: 10.2196/37559 (PMC9732752; doi:10.2196/37559)
Supplement: Multimedia Appendix 1 [file jmir_v24i11e37559_app1.pdf]

Appendix 1. Pretest and posttest survey items.

- **Pretest survey items**

Prior Knowledge

The following set of questions is about HPV. HPV stands for Human Papillomavirus. It is not HIV, HSV, or herpes. "*Before today, have you ever heard of...*"

1. HPV?
2. The HPV vaccine?

Yes (1) -- No (2) -- Don't know (3)

Behavioral intention at pre-test

[Display this question for participants with daughter(s) ages between 9 and 14]

The HPV vaccine requires two shots for 9 to 14 year-old children. Has your 9-14 year-old daughter been vaccinated for HPV? (If you have more than one daughter 9-14 years old, please answer about the daughter who had the most recent birthday.)

Yes, she has received two or more HPV vaccine shots (1)
Yes, she has received one HPV vaccine shot. (2)
No, she has not received any HPV vaccine shots. (3)
I don't know. (4)

[Display this question if: "No, he has not received any HPV vaccine shots" or "I don't know" is selected]

Thinking about the same 9-14 year-old daughter, how likely is it that she will receive the HPV vaccine in the next 12 months?

Very likely (1)
Somewhat likely (2)
Not sure/don't know (3)
Not too likely (4)
Not likely at all (5)

[Display this question for participants with son(s) ages between 9 and 14]

The HPV vaccine requires TWO shots for 9 to 14 year-old children. Has your *9-14 year-old son* been vaccinated for HPV? If you have *more than one son 9-14 years old*, please answer about the *son who had the most recent birthday.*

Yes, he has received two or more HPV vaccine shots (1)

Yes, he has received one HPV vaccine shot. (2)
No, he has not received any HPV vaccine shots. (3)
I don't know. (4)

[Display This Question if: "No, he has not received any HPV vaccine shots" or "I don't know" is selected.]

Thinking about the same 9-14 year-old son, how likely is it that he will receive the HPV vaccine in the next 12 months?

Very likely (1)
Somewhat likely (2)
Not sure/don't know (3)
Not too likely (4)
Not likely at all (5)

Attitudes toward the HPV vaccine at pre-test

Please indicate how you feel about *the HPV vaccine***.** For example, if you feel "*extremely good*" about the vaccine, click the bubble all the way on the right side as shown below.

"I would say the HPV vaccine is ... "

Bad (1)	--	Neutral (4)	--	Good (7)
Harmful (1)	--	Neutral (4)	--	Beneficial (7)
Foolish (1)	--	Neutral (4)	--	Wise (7)
Useless (1)	--	Neutral (4)	--	Useful (7)
Unpleasant (1)	--	Neutral (4)	--	Pleasant (7)
Unsafe (1)	--	Neutral (4)	--	Safe (7)
Unnecessary (1)	--	Neutral (4)	--	Necessary (7)
Concerning (1)	--	Neutral (4)	--	Not Concerning (7)
Not effective (1)	--	Neutral (4)	--	Effective (7)

Response efficacy

How effective do you think the HPV vaccine is in preventing…
- cervical cancer?
- mouth/throat cancer?

Extremely effective (5) -- Very effective (4) -- Fairly effective (3) -- Slightly effective (2) -- Not at all effective (1) -- I don't know (0)

- **Posttest survey items**

Manipulation check

1. The messages I saw were trying to promote HPV vaccine. Click your answer below.

Yes (1)
No (2)
I don't know (3)

2. Had you previously seen this message on television or other media?

Yes (1)
No (2)
I don't know (3)

Behavioral intention at post-test

Thinking about the same 9-14 year-old daughter/9-14 year-old son as before, how likely is it that she will receive the HPV vaccine in the next 12 months?

Very likely (1)
Somewhat likely (2)
Not sure/don't know (3)
Not too likely (4)
Not likely at all (5)

Post-test message perception 1: Message sensation value questionnaire

We would like you to rate the ads message you just saw on the following scales. Please indicate the degree to which you felt while viewing the ad.

For example, on the first pair of adjectives if you thought the ad was very "powerful impact" give a "1." If you thought it was very "Weak impact", give it a "7." If you thought it was somewhere in between, give it a 2, 3, 4, 5, or 6.

"I would say *the message that I just watched or read* is ... "

Powerful impact (1)	---	Neutral (4)	--	Weak impact (7)
Novel (1)	--	Neutral (4)	--	Ordinary (7)
Very convincing (1)	--	Neutral (4)	--	Not convincing (7)
Very helpful (1)	--	Neutral (4)	--	Not helpful at all (7)
Engaging (1)	--	Neutral (4)	--	Not engaging at all (7)

Post-test message perception 2: Perceived message effectiveness questionnaire

Please indicate how you felt about HPV vaccination after viewing the message.

1. How much did you find the message believable?
2. How much the message grabbed your attention?
3. How much the message made you want to consider HPV for your child(ren)?
4. Did the message convince you to change your opinion about the vaccine?

Not at all (1) --- Neutral (3) --- Very much (5)

Attitudes toward the HPV vaccine at post-test

After reading the message… "I would say the HPV vaccine is ... "

Bad (1)	--	Neutral (4)	--	Good (7)
Harmful (1)	--	Neutral (4)	--	Beneficial (7)
Foolish (1)	--	Neutral (4)	--	Wise (7)
Useless (1)	--	Neutral (4)	--	Useful (7)
Unpleasant (1)	--	Neutral (4)	--	Pleasant (7)
Unsafe (1)	--	Neutral (4)	--	Safe (7)
Unnecessary (1)	--	Neutral (4)	--	Necessary (7)
Concerning (1)	--	Neutral (4)	--	Not Concerning (7)
Not effective (1)	--	Neutral (4)	--	Effective (7)

Response efficacy at post-test

After reading the message, how effective do you think the HPV vaccine is in preventing…

1…. cervical cancer?
2. … mouth/throat cancer?

Extremely effective (5) -- Very effective (4) -- Fairly effective (3) -- Slightly effective (2) -- Not at all effective (1) -- I don't know (0)
