# Supplementary material for: Countering Antivax Misinformation via Social Media: Message-Testing Randomized Experiment for Human Papillomavirus Vaccination Uptake
Source: J Med Internet Res. 2022 Nov 24;24(11):e37559. doi: 10.2196/37559 (PMC9732752; doi:10.2196/37559)
Supplement: Multimedia Appendix 2 [file jmir_v24i11e37559_app2.pdf]

Appendix 2. Stimulus messages for the experimental group.

Theme 1: Countering 'Side Effects/Risk/Ingredient Concerns'

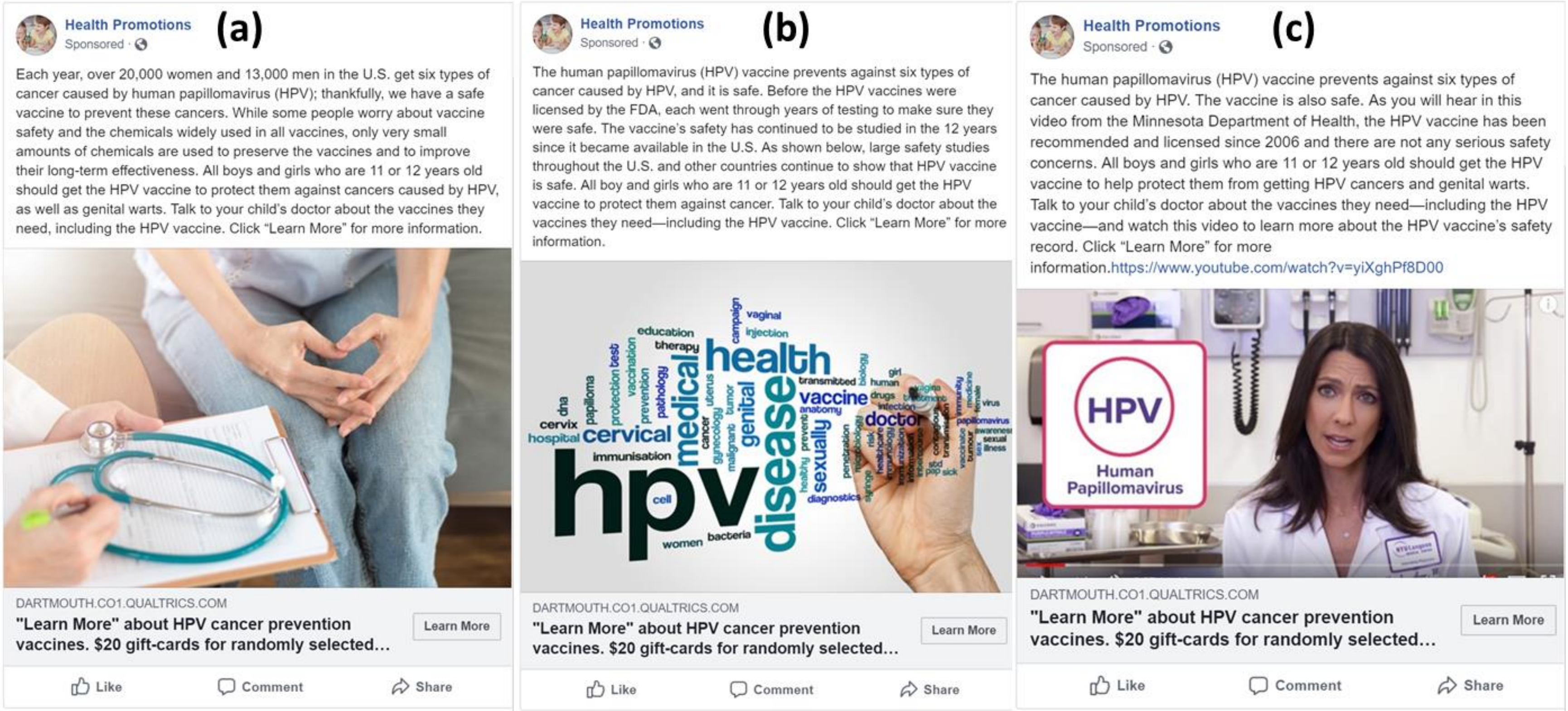

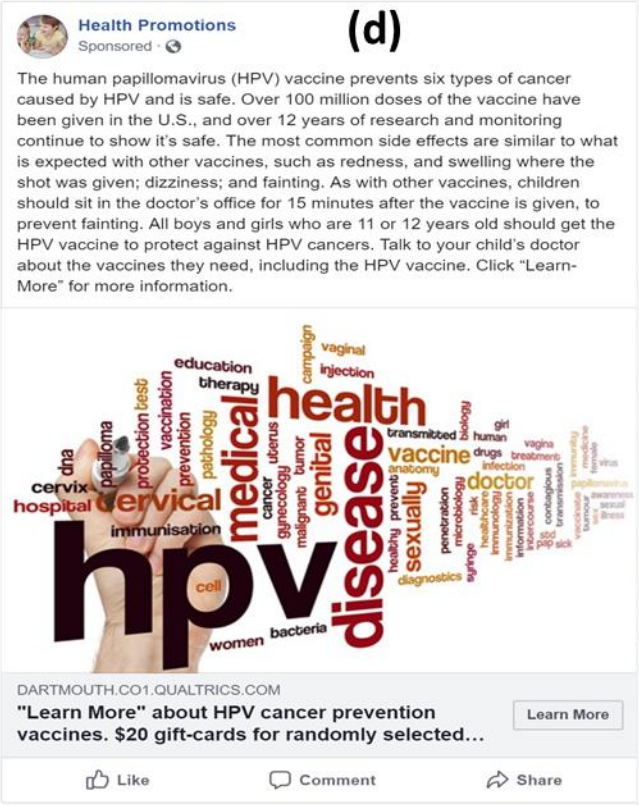
Health Promotions
Sponsored ·
(d)
The human papillomavirus (HPV) vaccine prevents six types of cancer caused by HPV and is safe. Over 100 million doses of the vaccine have been given in the U.S., and over 12 years of research and monitoring continue to show it's safe. The most common side effects are similar to what is expected with other vaccines, such as redness, and swelling where the shot was given; dizziness; and fainting. As with other vaccines, children should sit in the doctor's office for 15 minutes after the vaccine is given, to prevent fainting. All boys and girls who are 11 or 12 years old should get the HPV vaccine to protect against HPV cancers. Talk to your child's doctor about the vaccines they need, including the HPV vaccine. Click "Learn-More" for more information.
hpv
disease
health
medical
cervical
vaccine
doctor
genital
sexually
education
therapy
immunisation
hospital
cervix
DARTMOUTH.CO1.QUALTRICS.COM
"Learn More" about HPV cancer prevention vaccines. $20 gift-cards for randomly selected...
Learn More
Like
Comment
Share

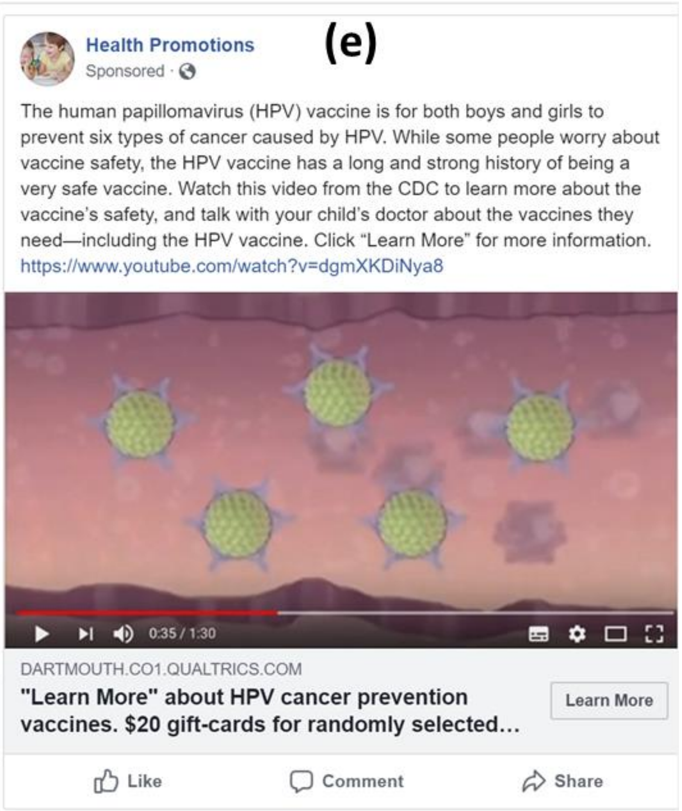
Health Promotions
Sponsored ·
(e)
The human papillomavirus (HPV) vaccine is for both boys and girls to prevent six types of cancer caused by HPV. While some people worry about vaccine safety, the HPV vaccine has a long and strong history of being a very safe vaccine. Watch this video from the CDC to learn more about the vaccine's safety, and talk with your child's doctor about the vaccines they need—including the HPV vaccine. Click "Learn More" for more information.
https://www.youtube.com/watch?v=dgmXKDiNya8
0:35 / 1:30
DARTMOUTH.CO1.QUALTRICS.COM
"Learn More" about HPV cancer prevention vaccines. $20 gift-cards for randomly selected...
Learn More
Like
Comment
Share

Theme 2: Countering 'Distrust of the System'

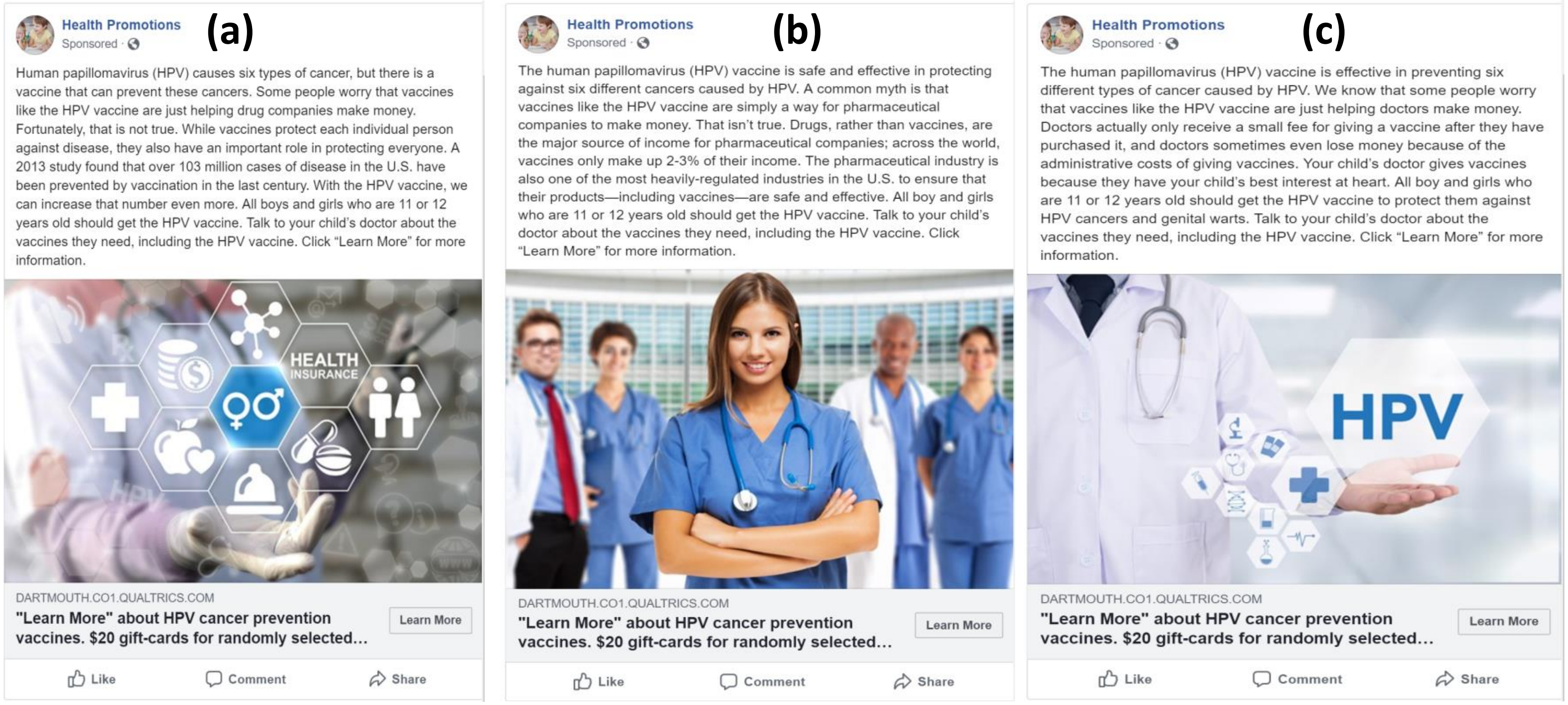

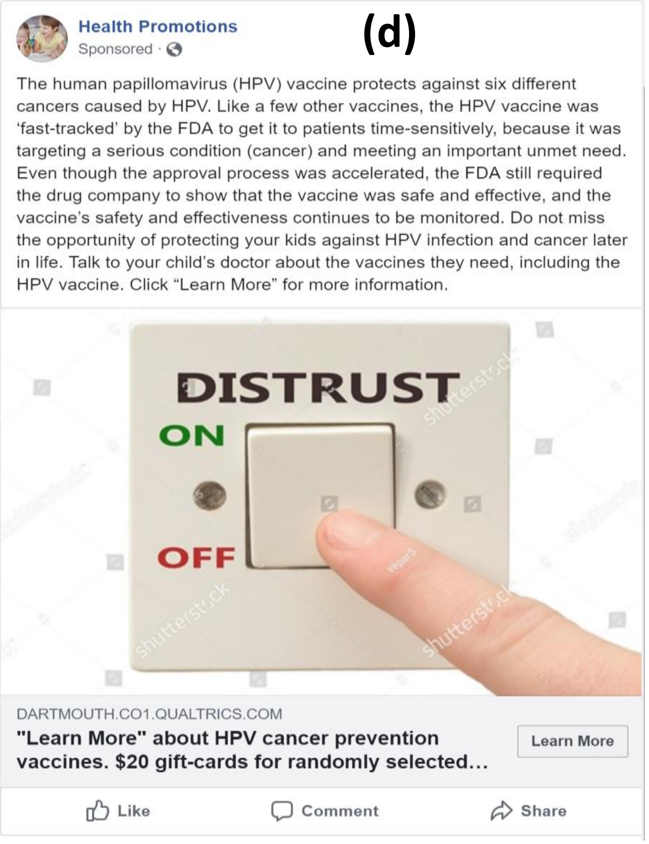
(d)
Health Promotions
Sponsored ·
The human papillomavirus (HPV) vaccine protects against six different cancers caused by HPV. Like a few other vaccines, the HPV vaccine was 'fast-tracked' by the FDA to get it to patients time-sensitively, because it was targeting a serious condition (cancer) and meeting an important unmet need. Even though the approval process was accelerated, the FDA still required the drug company to show that the vaccine was safe and effective, and the vaccine's safety and effectiveness continues to be monitored. Do not miss the opportunity of protecting your kids against HPV infection and cancer later in life. Talk to your child's doctor about the vaccines they need, including the HPV vaccine. Click "Learn More" for more information.
DISTRUST
ON
OFF
DARTMOUTH.CO1.QUALTRICS.COM
"Learn More" about HPV cancer prevention vaccines. $20 gift-cards for randomly selected...
Learn More
Like
Comment
Share

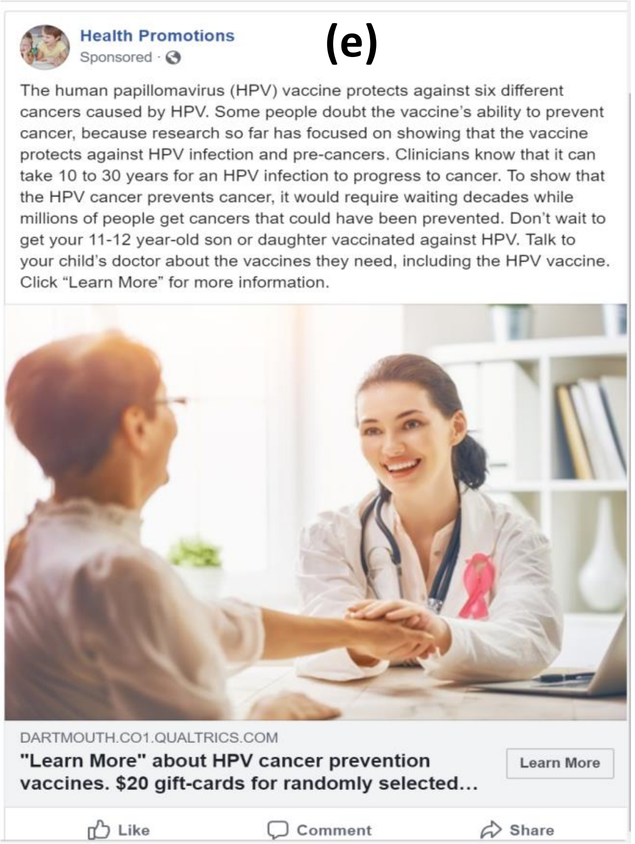
(e)
Health Promotions
Sponsored ·
The human papillomavirus (HPV) vaccine protects against six different cancers caused by HPV. Some people doubt the vaccine's ability to prevent cancer, because research so far has focused on showing that the vaccine protects against HPV infection and pre-cancers. Clinicians know that it can take 10 to 30 years for an HPV infection to progress to cancer. To show that the HPV cancer prevents cancer, it would require waiting decades while millions of people get cancers that could have been prevented. Don't wait to get your 11-12 year-old son or daughter vaccinated against HPV. Talk to your child's doctor about the vaccines they need, including the HPV vaccine. Click "Learn More" for more information.
DARTMOUTH.CO1.QUALTRICS.COM
"Learn More" about HPV cancer prevention vaccines. $20 gift-cards for randomly selected...
Learn More
Like
Comment
Share

Theme 3: Countering 'Effectiveness Concerns'

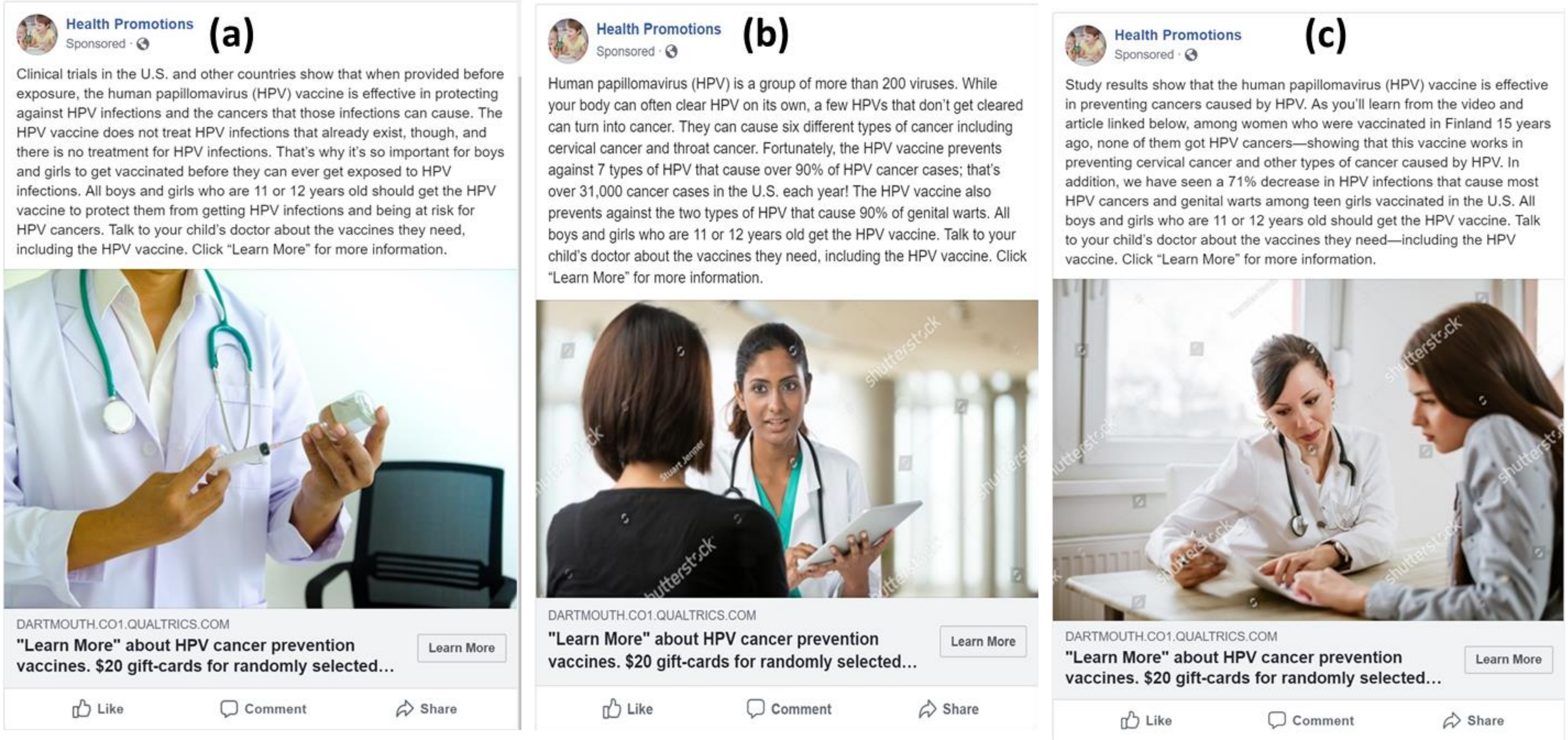

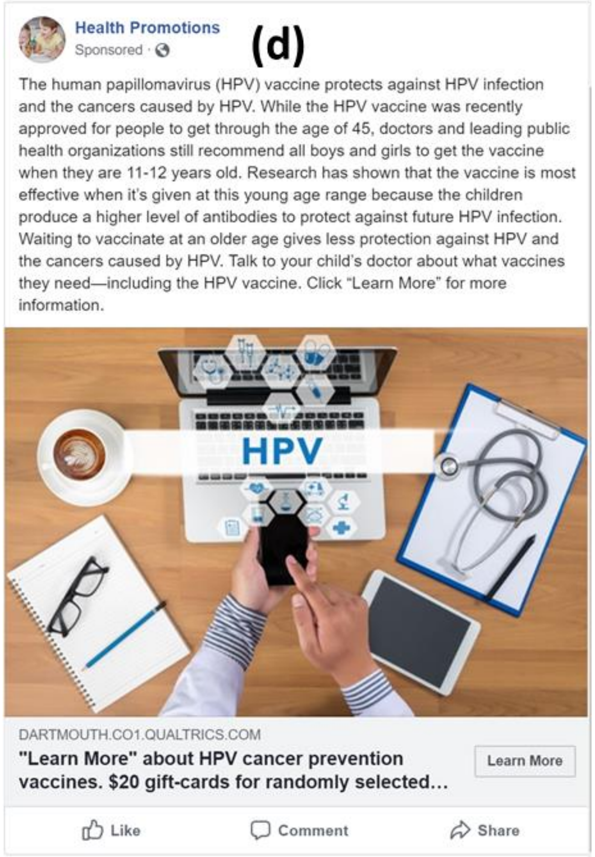
Health Promotions
Sponsored
(d)
The human papillomavirus (HPV) vaccine protects against HPV infection and the cancers caused by HPV. While the HPV vaccine was recently approved for people to get through the age of 45, doctors and leading public health organizations still recommend all boys and girls to get the vaccine when they are 11-12 years old. Research has shown that the vaccine is most effective when it's given at this young age range because the children produce a higher level of antibodies to protect against future HPV infection. Waiting to vaccinate at an older age gives less protection against HPV and the cancers caused by HPV. Talk to your child's doctor about what vaccines they need—including the HPV vaccine. Click "Learn More" for more information.
HPV
DARTMOUTH.CO1.QUALTRICS.COM
"Learn More" about HPV cancer prevention vaccines. $20 gift-cards for randomly selected...
Learn More
Like
Comment
Share

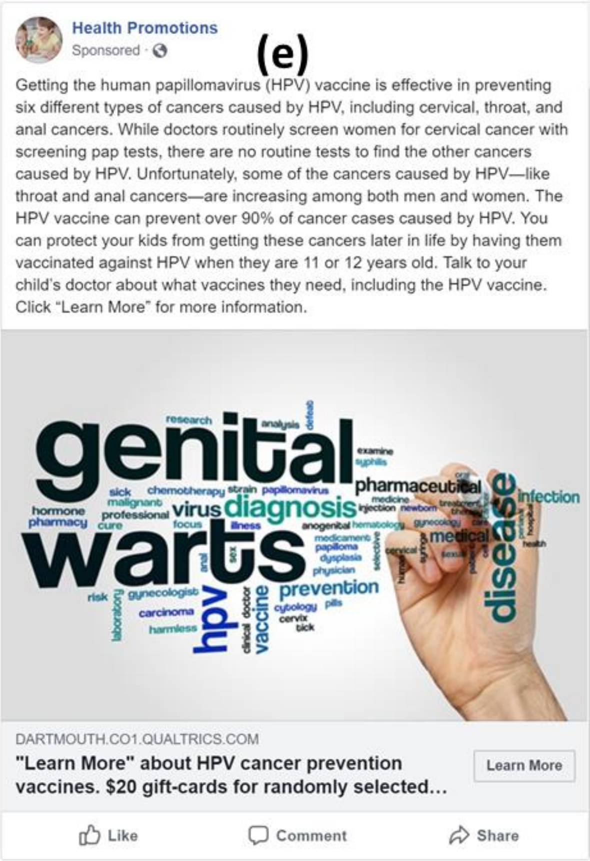
Health Promotions
Sponsored
(e)
Getting the human papillomavirus (HPV) vaccine is effective in preventing six different types of cancers caused by HPV, including cervical, throat, and anal cancers. While doctors routinely screen women for cervical cancer with screening pap tests, there are no routine tests to find the other cancers caused by HPV. Unfortunately, some of the cancers caused by HPV—like throat and anal cancers—are increasing among both men and women. The HPV vaccine can prevent over 90% of cancer cases caused by HPV. You can protect your kids from getting these cancers later in life by having them vaccinated against HPV when they are 11 or 12 years old. Talk to your child's doctor about what vaccines they need, including the HPV vaccine. Click "Learn More" for more information.
genital
warts
diagnosis
virus
prevention
hpv
vaccine
disease
pharmaceutical
infection
medical
DARTMOUTH.CO1.QUALTRICS.COM
"Learn More" about HPV cancer prevention vaccines. $20 gift-cards for randomly selected...
Learn More
Like
Comment
Share

Theme 4: Countering 'Connection to Sexual Activity'

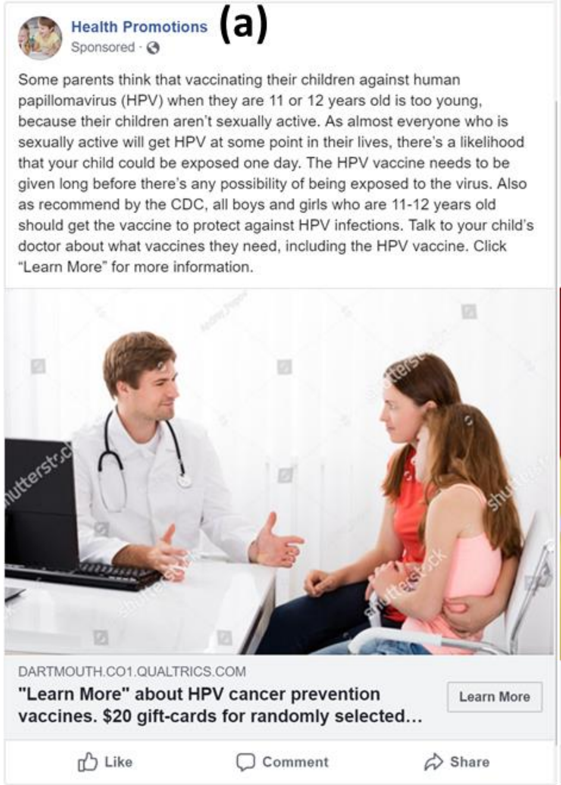

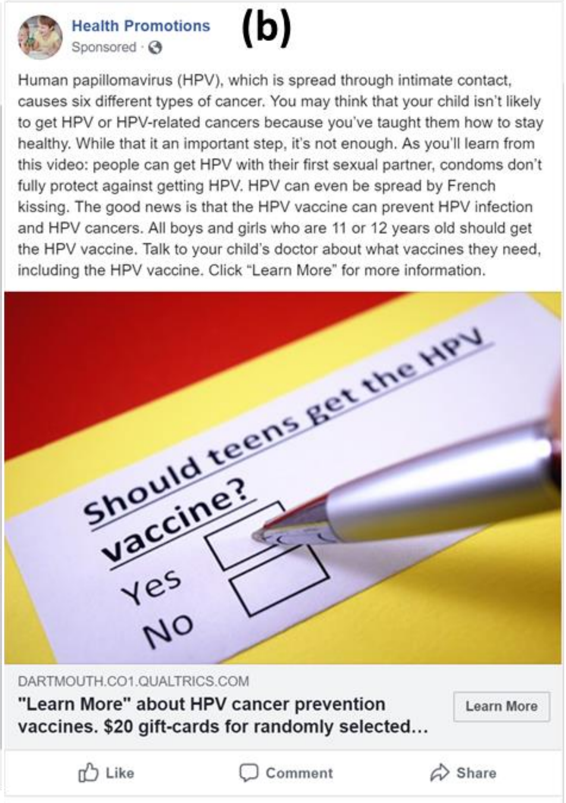

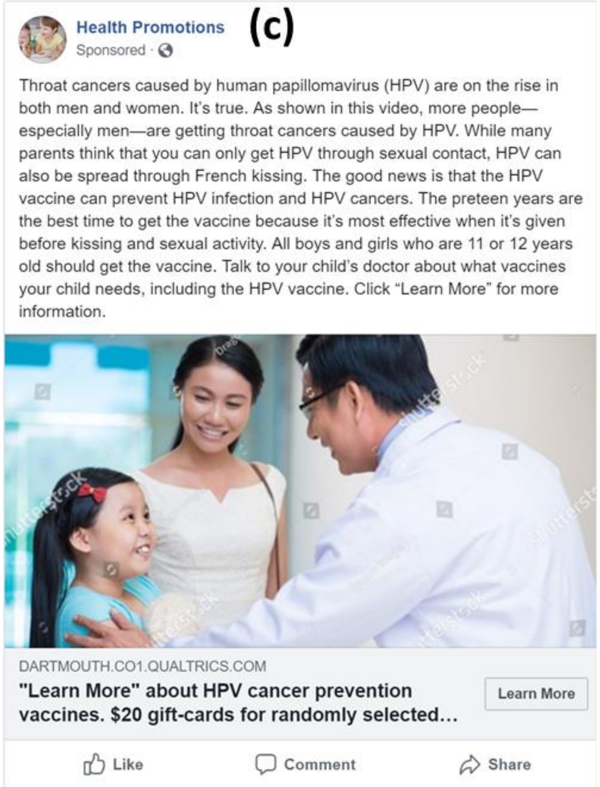

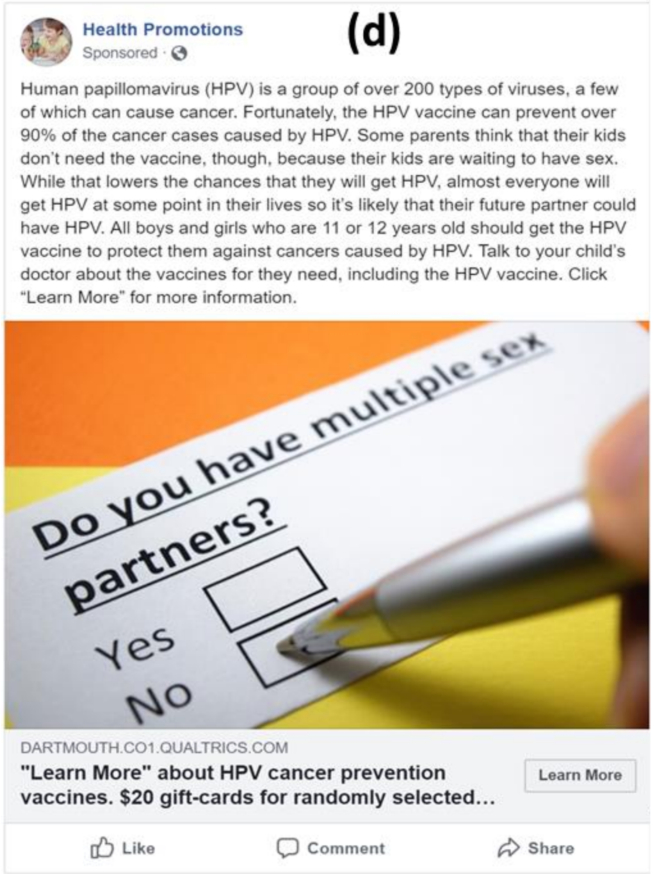
Health Promotions
Sponsored
(d)
Human papillomavirus (HPV) is a group of over 200 types of viruses, a few of which can cause cancer. Fortunately, the HPV vaccine can prevent over 90% of the cancer cases caused by HPV. Some parents think that their kids don't need the vaccine, though, because their kids are waiting to have sex. While that lowers the chances that they will get HPV, almost everyone will get HPV at some point in their lives so it's likely that their future partner could have HPV. All boys and girls who are 11 or 12 years old should get the HPV vaccine to protect them against cancers caused by HPV. Talk to your child's doctor about the vaccines for they need, including the HPV vaccine. Click "Learn More" for more information.
Do you have multiple sex partners?
Yes
No
DARTMOUTH.CO1.QUALTRICS.COM
"Learn More" about HPV cancer prevention vaccines. $20 gift-cards for randomly selected...
Learn More
Like
Comment
Share

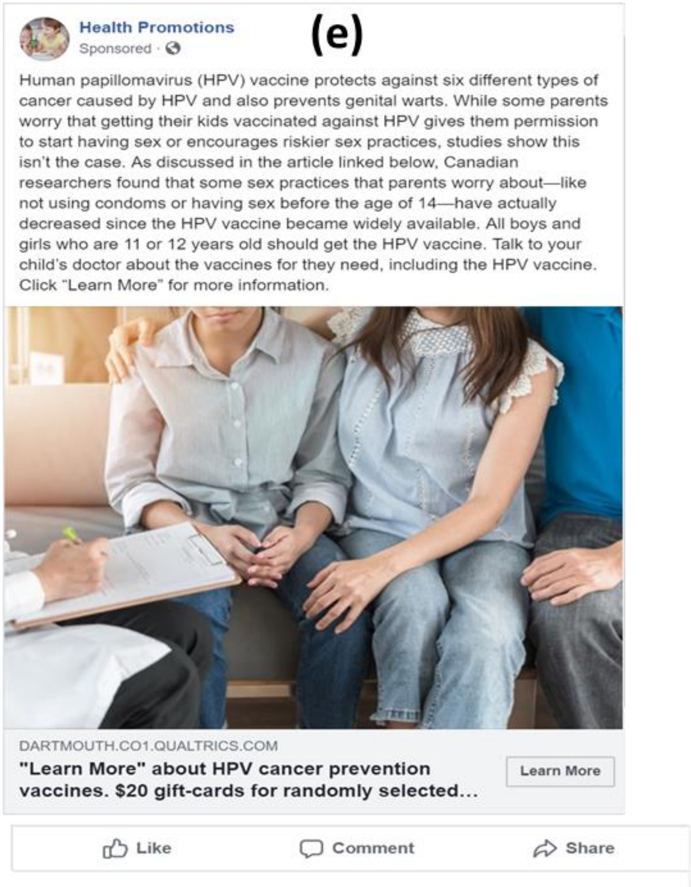
Health Promotions
Sponsored
(e)
Human papillomavirus (HPV) vaccine protects against six different types of cancer caused by HPV and also prevents genital warts. While some parents worry that getting their kids vaccinated against HPV gives them permission to start having sex or encourages riskier sex practices, studies show this isn't the case. As discussed in the article linked below, Canadian researchers found that some sex practices that parents worry about—like not using condoms or having sex before the age of 14—have actually decreased since the HPV vaccine became widely available. All boys and girls who are 11 or 12 years old should get the HPV vaccine. Talk to your child's doctor about the vaccines for they need, including the HPV vaccine. Click "Learn More" for more information.
DARTMOUTH.CO1.QUALTRICS.COM
"Learn More" about HPV cancer prevention vaccines. $20 gift-cards for randomly selected...
Learn More
Like
Comment
Share

Theme 5: Countering 'Misinformation'

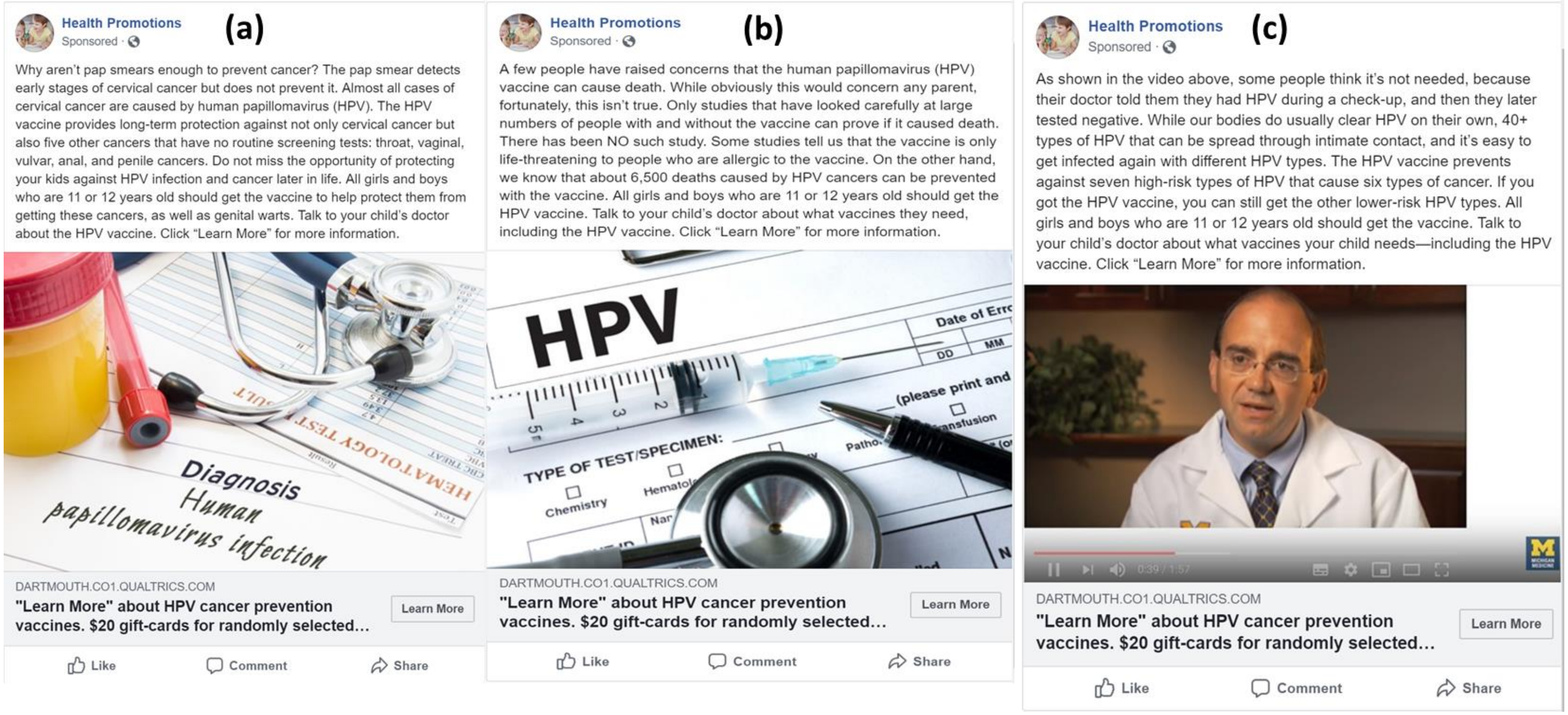

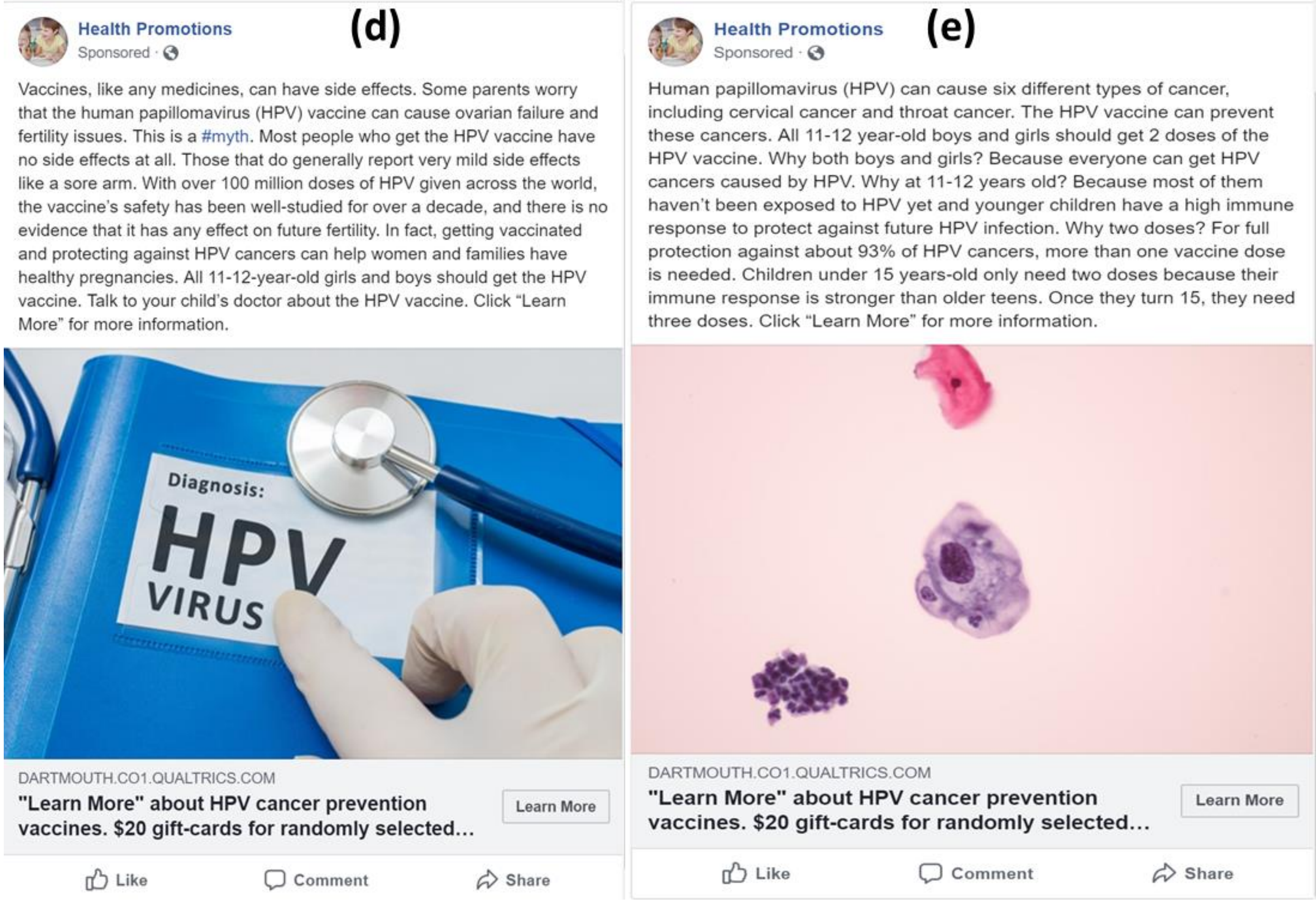
Health Promotions
Sponsored ·
(d)
Vaccines, like any medicines, can have side effects. Some parents worry that the human papillomavirus (HPV) vaccine can cause ovarian failure and fertility issues. This is a #myth. Most people who get the HPV vaccine have no side effects at all. Those that do generally report very mild side effects like a sore arm. With over 100 million doses of HPV given across the world, the vaccine's safety has been well-studied for over a decade, and there is no evidence that it has any effect on future fertility. In fact, getting vaccinated and protecting against HPV cancers can help women and families have healthy pregnancies. All 11-12-year-old girls and boys should get the HPV vaccine. Talk to your child's doctor about the HPV vaccine. Click "Learn More" for more information.
Diagnosis:
HPV
VIRUS
DARTMOUTH.CO1.QUALTRICS.COM
"Learn More" about HPV cancer prevention vaccines. $20 gift-cards for randomly selected...
Learn More
Like
Comment
Share
Health Promotions
Sponsored ·
(e)
Human papillomavirus (HPV) can cause six different types of cancer, including cervical cancer and throat cancer. The HPV vaccine can prevent these cancers. All 11-12 year-old boys and girls should get 2 doses of the HPV vaccine. Why both boys and girls? Because everyone can get HPV cancers caused by HPV. Why at 11-12 years old? Because most of them haven't been exposed to HPV yet and younger children have a high immune response to protect against future HPV infection. Why two doses? For full protection against about 93% of HPV cancers, more than one vaccine dose is needed. Children under 15 years-old only need two doses because their immune response is stronger than older teens. Once they turn 15, they need three doses. Click "Learn More" for more information.
DARTMOUTH.CO1.QUALTRICS.COM
"Learn More" about HPV cancer prevention vaccines. $20 gift-cards for randomly selected...
Learn More
Like
Comment
Share
